# The response of intercellular adhesion molecule-1 to exhaustive submaximal exercise and its correlation with physiological and anthropometric measures

**Published:** 2018

**Authors:** Hossein Taheri Chadorneshin, Ali Golestani, Fatemeh Jamali, Sakineh Mokhtari Motameni Shirvan, Hadi Sarir, Seyed Hosein Abtahi Eivary

**Affiliations:** * Department of Sport Sciences, University of Bojnord, Bojnord, Iran; ** Department of Animal Sciences, University of Birjand, Birjand, Iran; *** Department of Clinical Biochemistry, Gonabad University of Medical Sciences, Gonabad, Iran

**Keywords:** Exhaustive submaximal exercise, Intercellular adhesion molecule-1, Maximal oxygen consumption, Body mass index, Body fatpercentage, Waist-Hip ratio

## Abstract

**Introduction:**Intercellular adhesion molecule-1 (ICAM-1) acts as the main factor in the occurrence of atherosclerosis and inflammatory diseases. This study aimed to investigate the response of ICAM-1to exhaustive submaximal exercise and its correlation with maximal oxygen consumption (VO2max), body mass index (BMI), waist-hip ratio (WHR), body fat percentage (BF %) and calories burned during exercise (CB) in healthy men.

**Materials and methods:**Thirteen healthy men (mean ± standard deviation: age 23 ± 3 years, weight 78 ± 4 kg, height 180 ± 4 cm) cooperated in this quasi-experimental study and performed a single bout of exhaustive submaximal exercise on a cycle ergometer. Blood samples were collected from the antecubital vein before and immediately after exercise. Data were analyzed using the paired sample t-test and Pearson's correlation coefficient (α=0.05).

**Results:**Exhaustive submaximal exercisehad no significant effect on serum sICAM-1. Also, there were no significant correlations between ICAM-1 induced by the exhaustive submaximal exercise and VO2max, BMI, WHR, BF % and CB.

**Conclusions:**According to our findings, it cannot be ruled out that exhaustive submaximal exercise results in pathological and inflammatory conditions in healthy men. In Also, anthropometric and physiological parameters do not affect the response of ICAM-1 to exercise in healthy men.

## Introduction

Regular exercise training is one of the most important factors in the prevention and treatment of various diseases.Exercise training enhances well-being through several mechanisms (e.g., reduced fat and blood pressure, improved insulin sensitivity and artery function) [**1**]. However, exercise has shown to cause inflammatory conditions in the body [**2**]. Cell adhesion molecules (CAMs) act as main factors in inflammatory conditions [**2**][**3**][**4**].Studies have shown that cell-cell and cell-protein adhesions induced by CAMs have an important role in both physiological and pathological settings [**3**][**4**][**5**].

CAMs have been found as soluble and membrane-bound forms [**6**]. Membrane-bound intercellular adhesion molecule-1 (mICAM-1) with a molecular weight of 90 kDa is predominantly expressed on the surface of endothelial cells [**3**][**4**][**5**]. Furthermore, mICAM-1 are synthesized by keratinocytes, fibroblasts and granulocytes [**3**][**6**][**7**]. mICAM-1 acts as a receptor for membrane attachment of immune cells, especially leukocytes.Thereafter, leukocytes migrate into the tissue, activate endothelial cells, and ultimately accelerate the process of atherosclerotic plaque formation [**5**].Soluble intercellular adhesion molecule-1 (sICAM-1) is expressed and released into the circulation in response to adrenergic stimulation [**4**][**6**], inflammatory cytokines such as Interleukin 1 beta (IL-1β) and tumor necrosis factor alpha (TNF-α) [**3**][**8**][**9**], oxidative products [**6**][**8**][**10**][**11**][**12**] and shear stress induced by friction of blood against the vessel wall [**4**][**6**][**7**]. Collectively, soluble forms of ICAM-1 appear to reflect their expression on endothelial cells and play key roles in clinical and pathological conditions [**4**].

Although changes in high- and low-density lipoprotein (HDL and LDL, respectively) account as cardiovascular risk factors, studies have reported people who suffer from cardiovascular disease despite their normal levels of HDL-C and LDL-C [**1**][**5**]. Several studies have related cardiovascular diseases to inflammation. Therefore, more attention was paid to inflammatory factors for predicting cardiovascular disease. ICAM-1 is also used as a novel and critical tool for the diagnosis of vascular disorders and inflammatory conditions in humans [**1**][**3**][**5**]. Due to the important role of physical activity in the prevention of cardiovascular disease, researchers have studied the effects of exercise training on ICAM-1 [**1**][**4**][**5**]. As a result, a reduction in serum ICAM-1 has been reported in patients with coronary artery disease following a supervised exercise training program [**13**]. Also, a negative correlation has been reported between serum ICAM-1 and maximal oxygen consumption (VO2max), body mass index (BMI) and body fatpercentage (BF %) following exercise training [**14**]. In addition, an increase has been shown in ICAM-1 concentration following a single bout of wrestling practice session [**15**], long-distance running [**2**] and downhill running on a treadmill [**10**]. In contrast, no significant change in serum ICAM-1 concentration has been revealed in lean and obese participants following resistance exercise [**16**].

There is now a substantial body of evidence to suggest thatincreased levels of serum ICAM-1 are associated with the occurrence of atherosclerosis, myocardial infarction, peripheral artery disease and diabetes mellitus type 2 [**4**][**6**][**8**][**13**][**14**]. To protect against these conditions, it is important to monitor one’s serum ICAM-1 levels. There are contradictory findings regarding the effects of exercise on serum ICAM-1. In addition, the effects of intensive exercise on serum ICAM-1 levels have not yet been well examined. Moreover, studies have demonstrated that reactive oxygen species [**6**][**8**][**10**][**11**][**12**], inflammatory cytokines [**3**][**8**][**9**], and shear stress [**4**][**6**][**7**] increase the expression of adhesion molecules on endothelial cells. Intriguingly, it has been demonstrated that intensive exercise results in increasing levels of IL-1β, TNF-α [**17**], shear stress and subsequent oxidative stress [**18**] that mayexert a unique influence on the expression of ICAM-1.Thus, the concern arises whether ICAM-1 expression and subsequent individual susceptibility to inflammatory diseases are regulated by exhaustive exercise or not. Furthermore, is there any association between the anthropometric and physiological measures of subjects with serum ICAM-1 concentration induced by exhaustive submaximal exercise or not? Collectively, the result of the present study will provide new insight concerning the response of ICAM-1 to intensive exercise in healthy subjects.

## Materials and methods

**Subjects**

The present research was a quasi-experimental study with pre- and post-test on one group. Thirteen male volunteers (Shahid Beheshti University, Iran) were chosen after they completed the Physical Activity Readiness Questionnaire (PAR-Q). Also, physical fitness level was evaluated by Baecke’s Physical Activity Questionnaire.According to Bielinski and colleagues’ study (2008), our exclusion criteria included smoking, cholesterol-lowering medication, non-steroidal anti-inflammatory drugs, cardiovascular diseases, atherosclerosis, hyperlipidemia, hypertension, diabetes, and cancer [**19**]. All participants signed awritten informed consent knowing the potential benefits and the study-associated risks. The anthropometric and physiological measures of the subjects are depicted in **Table 1**.

**Table 1:** The anthropometric and physiological measures of the subjects

Age (yr)	23 ± 3
Weight (kg)	78 ± 4
Height (cm)	180 ± 4
BMI (kg/m^2^)	24 ± 3
VO_2_max, ml.kg^-1^.min^-1^	40.02 ± 4
BF %	15.46 ± 2
The values are mean ± standard deviation. Abbreviations: BMI, Body mass index; VO2max, Maximal oxygen consumption; BF %, Body fat percentage; WHR, waist-hip ratio.	

**Anthropometric measures**

All anthropometric measures were evaluated by the same examiner before performing the exhaustive submaximal exercise. The subject's height and weight were measured by the Seca digital stadiometer. BMI was calculated as weight in kilograms divided by the square of height in meters [**20**]. WHR was calculated as waist measurement (taken at the smallest circumference of the waist, just above the navel) divided by the hip measurement (the widest part of the buttocks) [**20**]. Skinfold thickness was obtained using skinfold caliper (Slim Guide model, USA) on the right side of the subject’s body. The Jackson-Pollock 3-site skinfold equation (chest, abdomen and thigh) was used to estimate body density, and BF % was subsequently calculated using the Siri equation [**20**].

**VO_2_max measure**

To evaluate theVO2max, the individuals were asked to cycle on a Monark electronically braked cycle ergometer (Model Ergomedic 839E,Varberg, Sweden), while respiratory gas exchange data were assessed using an online gas analyzer (Metalyzer 3B; Cortex Biophysik Gmbh; Leipzig; Germany).The test began with a 5-min warm-up without load.After the warm-up, the workload was increased to 50 watts for 2 minutes and then to 25 watts every minute until volitional fatigue. Volunteers were verbally encouraged to continue as long as possible [**21**]. They were asked not to workout exhaustively in the previous 48 hours.The criterion used to assess VO2max included heart rate at 90% of the age-predicted maximum, a respiratory exchange ratio higher than 1.1 and a plateau in oxygen uptake despite a further increase in workload. At least two of the three criteria were enough to stop the protocol [**21**].

**Exhaustive submaximal exercise**

The subjects were instructed to perform an exhaustive submaximal exercise on a Monark electronically braked cycle ergometer (Model Ergomedic 839E, Varberg, Sweden), five days after VO2max was determined [**21**]. During the first 20 minutes, the subjects cycled at 50 % of VO2max (at 60 pedal strokes per min), after which the work rate was increased to 65 % VO2max (at 60 pedal strokes per min) for a further 40 minutes. Finally, the subjects were instructed to cycle at their highest tolerable work rates to the point of exhaustion [**22**].At the end of the exhaustive exercise, the number of calories burned (CB) was recorded on the cycle ergometer for each subject.

**Blood sampling and biochemistry assay**

Blood samples (3 ml) were collected by the same nurse from the antecubital vein before and immediately after the exhaustive submaximal exercise. The samples were centrifuged (Eppendorf Centrifuge, Mini SpinR, Germany) for 10 min at 3000 ×g, at 4 °C. Serums were collected and stored immediately at −80 °C. The ICAM-1 level in the serum was measured by a biochemistry expert using the commercial ELISA kit (Catalogue numbers: 850.540.096, Gen-Probe Diaclone SAS, France) according to the manufacturer’s instructions.The absorbance of ICAM-1 was measured at 450 nm using the Anthos 2020 microplate reader (Biochrom CO, England). The sensitivity, minimum detectable dose of ICAM-1, was found to be less than 0.1 ng/ml.The intra- and inter-assay coefficients of variation were calculated to be 1.03 % and 3.93 %, respectively. The pre- and post-test reliability of serum ICAM-1 was examined using the interclass coefficient correlation (ICC). A significant correlation (0.67) was observed.

**Statistical analysis**

Data are presented as mean ± standard deviation and analyzed using the Statistical Package for Social Sciences (SPSS Inc., Chicago, USA) software, version 16.0. Initially, normality and equality of variances were approved by Shapiro-Wilk's and Levene's tests, respectively. Statistical analysis was carried out using paired-samples t-test and Pearson's correlation coefficient at p < 0.05.

## Results

**Fig. 1:**
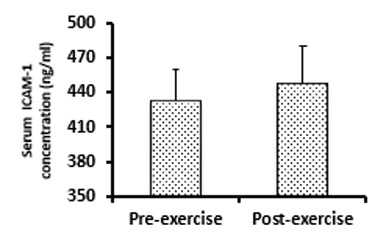
A single bout of exhaustive submaximal exercise had no significant effect on serum ICAM-1 in healthy men.

**Fig. 2:**
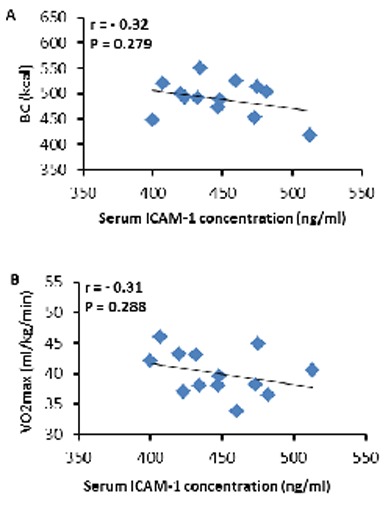
There were no significant correlations between serum ICAM-1 induced by the exhaustive submaximal exercise and physiological measures, i.e., BC (A) and VO2max (B).

**Fig. 3:**
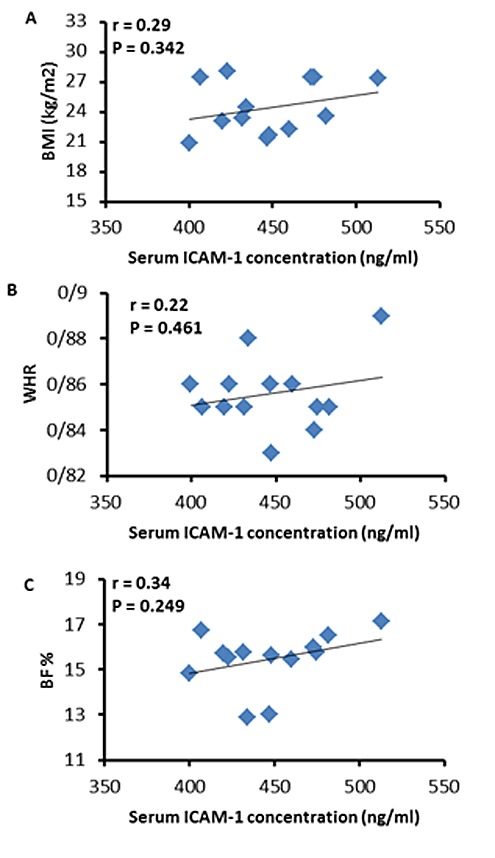
There were no significant correlations between serum ICAM-1 induced by the exhaustive submaximal exercise and anthropometric measures, i.e., BMI (A), WHR (B) and BF % (C).

The average time of the exhaustive submaximal exercise tovolitional fatigue (exhaustion) was 69 ± 8 min.Also, the rate of calories burned (CB) during exercise was 490 ± 36 kcal. A single bout of exhaustive submaximal exercise did not have a significant effect on serum ICAM-1 immediately after cycling (447.28 ± 32.60 ng/ml) compared to the baseline level (432.22 ± 27.45 ng/ml) (t12 = 1.60, P = 0.13) (**Fig. 1**). Furthermore, our results showed no significant correlations between serum ICAM-1 induced by exhaustive submaximal exercise and CB (r = - 0.32, P = 0.279)(**Fig. 2**A), VO2max (r = - 0.31, P = 0.288)(**Fig. 2**B), BMI (r = 0.29, P = 0.342)(**Fig. 3**A), WHR (r = 0.22, P = 0.461)(**Fig. 3**B), and BF % (r = 0.34, P = 0.249)(**Fig. 3**C).

## Discussion

Although changes in the lipid profile account as cardiovascular risk factors, studies have reported people who suffer from cardiovascular disease despite their normal levels of the lipid profile [**1**][**5**]. A significant amount of research suggests that ICAM-1 plays a pivotal role in the development of atherosclerosis [**1**][**3**][**5**]. It has been showed that shear stress [**4**][**6**][**7**], reactive species [**6**][**8**][**10**][**11**][**12**], and inflammatory markers [**3**][**8**][**9**] increase the expression of ICAM-1. On the other hand, one bout of exercise with maximum effort increases reactive species [**18**] and inflammatory markers [**17**] that may result in expression of ICAM-1. However, the results of the present study showed no significant change in serum ICAM-1 followed by exhaustive submaximal exercise.

Our observations align with the reports [**16**][**23**] showing that exhaustive submaximal exercise has no significant effect on serum ICAM-1. In this regard, it has been showed that resistance exercise (3 sets of 10 resistance exercises with 10–12 repetitions at 70–75% of one-repetition maximum) [**16**] and cycling for 90 min at 65% of the VO2max [**23**] did not affect the serum concentration of ICAM-1 in lean [**16**][**23**] and obese [**16**] participants. The lack of change in serum ICAM-1 following resistance exercise may be due to increased levels of anti-inflammatory factors and decreased levels of inflammatory factors. An increase in IL-6 and a reduction in IL-1β have been reported following high-intensity eccentric exercise (bench press and leg curl at an intensity equal to 100% of the one-repetition maximum) [**24**]. There is a transient increase in circulating levels of anti-inflammatory cytokines (IL-6 and adiponectin) following acute exercise that inhibits ICAM-1 expression induced by TNF-α, whereas chronic exercise reduces basal levels of pro-inflammatory cytokines [**25**]. Our findings are inconsistent with other studies because of the clinical status of their participants (subjects with coronary arthritis and diabetes) and their exercise training protocol (supervised exercise training program) that resulted in a reduction in serum ICAM-1 [**13**][**14**]. A high level of serum ICAM-1 has been reported in coronary arthritis and diabetes patients [**4**]. However, it is known that exercise training reduces and improves coronary arthritis and diabetes [**1**]. So, part of the reduction in serum ICAM in these patients may be due to the improved disease following exercise [**13**][**14**]. Especially, Rector and colleagues have reported that exercise improves vascular function and thereby reduces the serum levels of ICAM1 [**20**]. On the contrary, an increased level of serum ICAM-1 has been indicated following 1.5 hours of intensive wrestling in adolescent boys [**15**]. Also, increased plasma level of ICAM-1 has been demonstrated in men participating in the Oslo marathon [**2**]. Therefore, increased levels of ICAM-1 may be due to the long duration of the long-distance running marathon, because the duration of cycling in our study was lower compared to other studies [**2**]. Exercise with high intensity and long duration lead to increased production of reactive oxygen species (ROS) and oxidation of LDL. Oxidized lipoprotein increases the expression of inflammatory markers, particularly ICAM-1 [**6**]. Also, Akimoto and colleagues (2002) have reported increased levels of ICAM-1 one day after a 30-min downhill running at the subjects’ ventilation threshold [**10**]. Muscle damage induced by downhill running increases neutrophils and leukocytes migration to the damaged area 12 hours after the muscle damage and macrophages produce free radicals, thereby causing inflammatory conditions and increasing ICAM-1 plasma levels [**4**][**10**][**12**]. In this regard, it has been proved that antioxidant supplementation (particularly vitamin E) reduces the expression of ICAM-1 on endothelial cells by direct scavenging of ROS, reduced monocyte binding to endothelial cells, reduced release of pro-inflammatory cytokines such as TNF-α and IL-1β, and increased resistance of LDL to oxidation [**6**][**11**]. Collectively, it seems that the duration of exercise [**2**], muscle damage [**4**][**10**][**12**] and production of ROS [**6**][**8**][**11**] influence ICAM-1 levels.

Our study showed a negative and nonsignificant correlation between serum ICAM-1 induced by exhaustive submaximal exercise and VO2max and calories burned. Our findings suggest that reduced serum levels of ICAM-1 correspond to the increased aerobic power and amount of calories burned during exhaustive exercise. It appears that subjects with high aerobic power have a large vascular network that subsequently affects the response of endothelial cells to shear stress [**8**]. In reality, the low response of endothelial cells to shear stress results in low expression of ICAM-1 on endothelial cell [[**4**][**6**][**7**]. Tonjes and colleagues (2007) have shown a negative correlation between ICAM-1 and VO2max after 4 weeks of intensive exercise training. This correlation was determined at the exercise protocol end-stage when subjects experienced increased levels of VO2max and reduced levels of ICAM-1 [**14**].This correlation may be due to angiogenesis induced by exercise training that resulted in reduced shear stress and ICAM-1 expression [**14**]. In addition, Adamopoulos et al., reported in a study a negative correlation between ICAM-1 and VO2max (r = - 0.72) inchronic heart failure patients following 12 weeks of cycling (at an intensity corresponding to 70 to 80% of the maximal heart rate) [**8**]. In these subjects, it was observed that VO2max was low, but serum levels of ICAM-1 were high. In other words, pathological condition and higher levels of ICAM-1 resulted in this negative correlation [**8**].

Finally, a non-significant correlation between serum ICAM-1 induced by exhaustive submaximal exercise and calories burned was found for the first time in the present study. In contrast to other reports [**9**][**23**], our findings showed no significant correlations between serum ICAM-1 after the exhaustive submaximal exercise and BMI, WHR, and BF % while another study reported the same result [**15**]. Nemet et al. (2004) demonstrated an increase in serum ICAM-1 following wrestling training in boys aged 14–18 years [**15**]. However, no correlation was found between fitness (peak VO2/kg), BMI and the magnitude of change in response of ICAM-1 to exercise [**15**].The subjects in their study were all relatively fit and their BMI may be determined by increased muscle mass rather than increased fat [**15**]. In this context, Pontiroli and colleagues (2004) revealed that change in serum ICAM-1 significantly correlated with BMI change in obese subjects. Also, they reported greater levels of ICAM-1 in obese compared to lean subjects [**9**]. Since some cytokines are secreted by the adipose tissue resulting in the expression of ICAM-1 on endothelial cells [**3**][**8**][**9**], it seems that obesity resulted in the significant relationship between the two variables in their investigation. Moreover, part of the significant correlation in the study of Pontiroli et al. may be due to the greater number of subjects (96). In contrast, the number of participants in our study was low (13 subjects). Despite the reduction in fat content and ICAM-1 levels, no significant correlation has been shown between changes in ICAM-1 and changes in BF% following lifestyle modification in sedentary, overweight adults [**20**]. It is thought that decreases in shear stress, independent of changes in body fat, reduce ICAM-1 levels [**20**].

In our study, exhaustive submaximal exercise resulted in only 6% increase in serum ICAM-1. Studies have shown that exhaustive exercise increases blood flow and subsequently shear stress [**26**][**27**][**28**]. Therefore, increased shear stress induced by the exhaustive submaximal exercise results in releasing of ICAM-1 from endothelial cells to the bloodstream [**4**][**6**][**7**]. Also, secretion of inflammatory cytokines such as TNF-α from adipose tissue increases during exercise that subsequently increases expression of ICAM-1 in endothelial cells [**3**][**8**][**9**][**12**]. In addition, intensive exercise increases the release of inflammatory cytokines from adipocytes, which subsequently increases expression of ICAM-1 on endothelial cells [**2**][**3**][**9**].

## Conclusion

Collectively, according to our findings, it cannot be ruled out that exhaustive submaximal exercise results in pathological and inflammatory conditions in healthy men.In addition, there is no association between the anthropometric and physiological measures of healthy men with ICAM-1 concentration induced by exhaustive submaximal exercise.

**Conflict of interest**

The authors declare that there is no conflict of interest.
